# Modeling and Sensitivity Analysis of GaN HEMT Biosensor

**DOI:** 10.3390/bios16090520

**Published:** 2026-09-17

**Authors:** Ashkhen Yesayan, Jean-Michel Sallese

**Affiliations:** STI-EDLab, Ecole Polytechnique Federal de Lausanne (EPFL), 1015 Lausanne, Switzerland

**Keywords:** GaN HEMT biosensor, MIS-HEMT biosensor, COMSOL simulations, analytical model

## Abstract

Gallium nitride (GaN) high-electron-mobility transistor (HEMT) biosensors have recently emerged as promising platforms for highly sensitive and label-free detection of biomolecules. Their exceptional electrical properties, including high carrier mobility, together with the wide bandgap and strong chemical bonds of GaN materials, provide excellent stability under high temperatures, ionizing radiation, and chemically harsh environments. Despite significant experimental progress, comprehensive analytical models capable of linking biomolecular recognition events to the electrical response of GaN biosensors remain limited. This work presents a physics-based, design-oriented analytical modeling framework for AlGaN/GaN HEMT biosensors. The model incorporates biomolecular binding kinetics, the dielectric properties of the hybrid system, and electrostatic coupling to the transistor channel conductivity. Numerical simulations are performed using COMSOL Multiphysics to validate the analytical model. The developed framework provides physical insight into the mechanisms governing biosensor operation and offers practical guidelines for the optimization and comparative assessment of HEMT/MIS-HEMT biosensor architectures.

## 1. Introduction

Recently, experimental data have demonstrated that GaN HEMTs are a breakthrough technology for biosensing applications, including clinical diagnosis, point-of-care testing, and on-site detection [1,2,3,4,5,6,7,8,9,10,11,12,13,14,15,16,17,18,19,20,21,22,23,24,25,26,27,28,29,30,31,32,33,34,35,36,37,38]. GaN HEMTs were originally developed as RF devices, and they inherently demonstrate low flicker noise, high cut-off frequency, and fast transient response. When introducing GaN HEMTs as biosensors, these characteristics enable faster detection kinetics and improved time-resolved sensing performance. The transition of GaN HEMT technology from power electronics to biosensing applications represents a significant convergence of semiconductor physics and biomedical engineering. Recent extensive research on GaN HEMT biosensors proves their potential for the early-stage detection of cancer cells and pathogens [2,3,4,5,6,7,8,9,10]. Highly sensitive immunological diagnostic methods that directly detect viral antigens in clinical samples without sample preparation steps are highly desirable. This explains the growing interest in further improving the sensitivity limits of existing field-effect biosensors. Appropriate functionalization of GaN HEMTs allows the detection of specific molecules, kidney injury markers [10], pH [11,12,30,37], proteins, and viral/bacterial biomolecules [9,12]. GaN HEMTs have been investigated as immuno-biosensors to measure the concentration of the stress hormone cortisol in human saliva [19]. GaN HEMT-based biosensors in lab-on-chip platforms can also be used for the prognosis of neurological disorders by detecting the Aβ biomarker in saliva samples [14]. They also have great potential for environmental monitoring, such as food safety detection and air/water pollution [17,31,38]. Finally, the compatibility of GaN HEMT fabrication with established semiconductor processing techniques enables high-density integration and miniaturization [14,15,33,36].

Compared with conventional silicon-based FET biosensors, GaN HEMT biosensors offer several advantages that make them particularly attractive for biochemical sensing applications. While silicon FET biosensors have been widely explored for healthcare applications owing to their compact size, CMOS compatibility, affordability, and high sensitivity, they suffer from limitations in chemical stability and reliability under extreme conditions.

The material properties of GaN are exceptionally suitable for operation in physiological environments. The strong ionic bonds in the GaN crystal lattice are resistant to chemical degradation, while the non-toxic nature of GaN facilitates its biocompatibility [23,24].

Heterojunction engineering of GaN-based HEMT biosensors pushes the device sensitivity to higher values compared with Si-based FET biosensors. The two-dimensional electron gas (2DEG) forms spontaneously within the quantum well (QW) at the AlGaN/GaN interface due to polarization effects inherent to the wurtzite crystal structure of these materials. This spontaneous formation of 2DEG due to polarization charges allows the use of an undoped thin barrier layer (typically 5–20 nm). This 2DEG channel is only a few nanometers from the sensing surface in GaN HEMT biosensors, making it highly sensitive to surface charge perturbations. Therefore, the high mobility of the 2DEG, the undoped AlGaN barrier, and the proximity of the channel to the surface enhance the response of HEMT biosensors and enable detection limits down to attomolar concentrations [3,21,22].

The combination of these advantages makes AlGaN/GaN HEMT biosensors interesting candidates for addressing the limitations of conventional biosensing technologies and meeting the growing demand for rapid, sensitive, and reliable detection platforms [20,25,26,33,34,35].

Despite the increasing number of experimental investigations, a comprehensive model of the GaN HEMT biosensor signal-transduction process is not available. Existing modeling approaches are mainly restricted to simple capacitance-based sensitivity estimations [24,25], while an SVR-based intelligent framework was proposed in [23] for optimization of the MIS-HEMT biosensor design space. The development of a physics-based, design-oriented, dynamic model linking biomolecular interactions to the electrical response of the device therefore remains a challenge. In this paper, we introduce an analytical model for the GaN HEMT/MIS-HEMT biosensor.

## 2. GaN HEMT Biosensor Operation and Modeling

### 2.1. The Structure of GaN HEMT Biosensor

**Figure 1:**
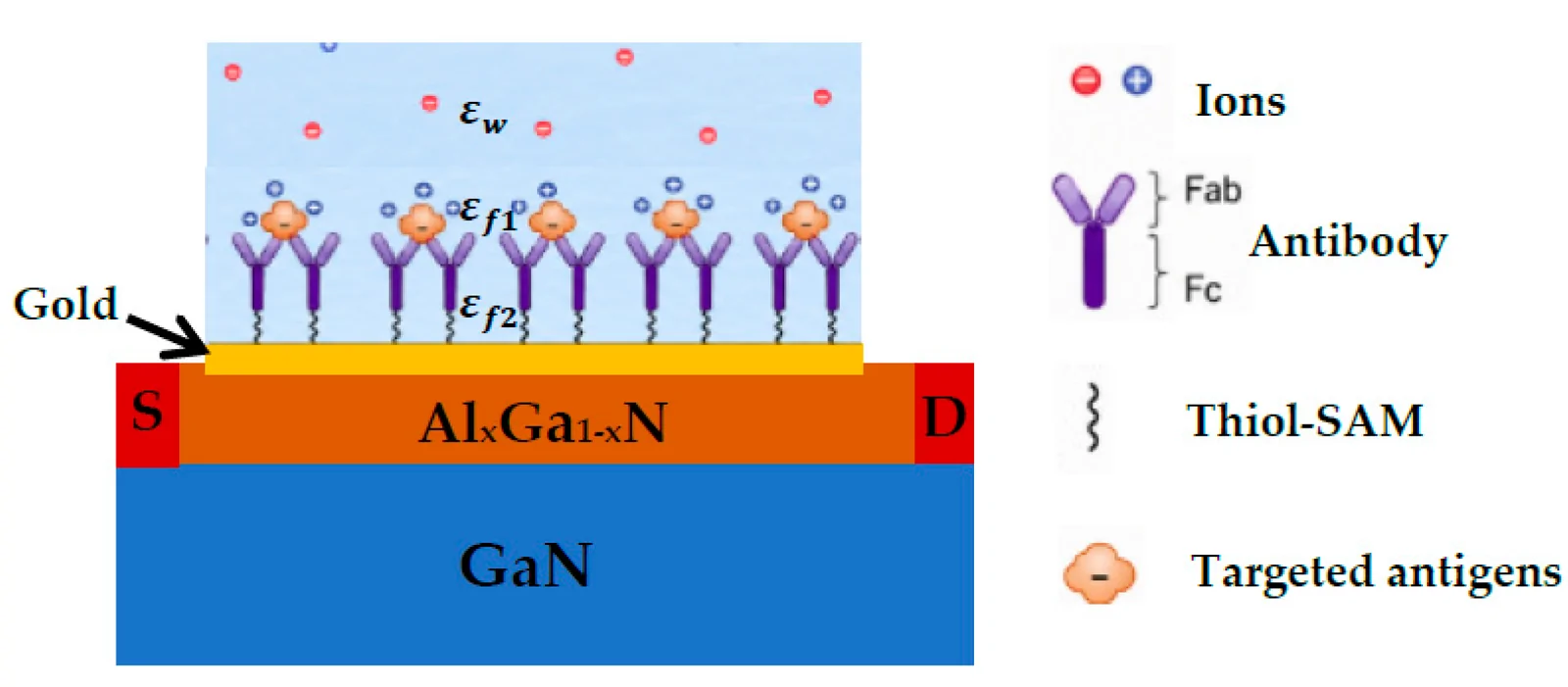
Typical structure of a GaN HEMT with a simplified representation of the functionalization and diffuse layers (not to scale).

The typical structure of a GaN HEMT-based biosensor is illustrated in Figure 1. The sensing surface is functionalized with a layer of biorecognition molecules, such as antibodies or aptamers, immobilized on the gold gate electrode. Upon target-analyte binding, the resulting surface charge modulation affects the electrical characteristics of the transistor. Depending on the presence of an insulating layer between the AlGaN barrier and the gate metal, the device is classified as either a Schottky-gate HEMT biosensor [2,9,15,19,32] or a MIS-HEMT biosensor [12,25]. In this work, we develop an analytical model for a GaN HEMT biosensor which can readily extended to MIS-HEMT biosensors.

### 2.2. Functionalized Layer and Biological Charge

The gold sensing surface is typically functionalized with a self-assembled monolayer (SAM) of thiol-containing molecules (R–SH), where the thiol group forms a strong bond with gold, while the terminal functional group serves as an attachment site for biorecognition elements. Upon biomolecular recognition, adsorption of the target analytes on the functionalized surface results in the formation of a biologically charged layer above the gate electrode. In this work, we consider antigens dispersed in the solution that bind specifically to antibodies immobilized on the GaN HEMT sensing surface. The formation of antigen–antibody (epitope–paratope) complexes generates an effective biological charge layer located above the AlGaN surface.

Unlike electrostatically induced charges, this biological charge originates from molecular binding events and is primarily governed by binding affinity, Gibbs free energy, and the chemical environment (e.g., pH). Consequently, the biological charge can be treated as independent of the gate potential. The resulting charge modulates the electrostatic potential at the sensing interface and, therefore, the two-dimensional electron gas (2DEG) concentration. This modulation alters the channel conductivity and drain current. Owing to the high electron mobility of the 2DEG, small variations in surface charge can produce significant electrical responses, enabling highly sensitive biosensing.

#### 2.2.1. Dynamics of the Biological Charge Density

The temporal evolution of the biological charge is determined by the adsorption kinetics of target biomolecules. Antibody–antigen binding is often considered effectively irreversible over the measurement timescale [39,40]. The overall sensing kinetics may be limited either by the adsorption reaction or by molecular diffusion. In the reaction-limited regime, molecular transport to the sensing surface is sufficiently fast compared with the adsorption reaction, such that the biomolecule concentration near the sensing surface remains close to its bulk value [41]. For the planar HEMT sensing surface, the characteristic depletion length can be estimated as $W\sim \sqrt{Dt}$ [41]. For a biomolecular diffusion coefficient of $D=10^{-6}\;{cm}^{2}s^{-1}$, the depletion length after t = 10^−2^ s is approximately of micrometer scale. This indicates that diffusion over micrometer-scale distances can occur on a timescale much shorter than the characteristic timescale of biochemical binding. Thus, the sensing is mainly reaction-controlled. To be more precise, the kinetic regime depends on the diffusion coefficient, characteristic depletion length, flow conditions, and adsorption kinetics. Although the properties of the immobilized receptors are not readily adjustable, the sensor dimensions and flow conditions can be engineered to favor the reaction-limited regime. For example, in blood or saliva analysis, where the available sample volume is generally not the primary constraint, transport conditions can be designed to favor the reaction-limited regime, allowing the measurement time to approach the timescale permitted by the underlying biochemical reaction [41].

Defining $Q_{bio}$ as the biological charge density bound to the sensing surface, the time-dependent charge can be expressed as [42]

(1) $$Q_{bio}\left(t\right)=q_{mol}N_{A}\Gamma_{max}(1-exp(-k_{ads}c_{o}t))$$

where $q_{mol}$ is the charge carried by a single biomolecule, $N_{A}$ is Avogadro number, $k_{ads}$ is the absorption coefficient, $\Gamma_{max}(mol/m^{2})$ is the density of active surface sites, and $c_{o}(M)$ is the species molar concentration. We assume that at a given time, the biological charge $Q_{bio}\left(t\right)$ (calculated from (1)) is bonded on the HEMT surface.

#### 2.2.2. Dielectric Permittivity of Functionalized Layer

The functionalization layer typically consists of a SAM of thiol-containing molecules and immobilized antibodies or aptamers. The antibody itself consists of Fab and Fc fragments (see Figure 1). We model the functionalized layer by considering different dielectric permittivities for the top layer, which consists of Fab regions of immobilized antibodies together with the bound antigens, and the deeper layer, which consists of Fc fragments of antibodies and the SAM layer. As discussed in [42], the upper region can be approximated as a suspension of spherical biomolecules embedded in an aqueous medium. Under this assumption, its effective permittivity can be estimated using the Maxwell–Garnett relation

(2) $$\varepsilon_{f1}=2\varepsilon_{w}\left(\eta_{bio}\frac{\varepsilon_{bio}-\varepsilon_{w}}{\varepsilon_{bio}+2\varepsilon_{w}}+1/2\right)/\left(1-\eta_{bio}\frac{\varepsilon_{bio}-\varepsilon_{w}}{\varepsilon_{bio}+2\varepsilon_{w}}\right)$$

where $\varepsilon_{f\_1}$ is the effective relative permittivity, $\varepsilon_{bio}$ is the relative permittivity of the biomolecules, $\varepsilon_{w}$ is the permittivity of the aqueous solution, and $\eta_{bio}$ is the volume fraction occupied by biomolecules.

The lower region consists of an aqueous medium interspersed with Fc fragments and SAM molecules. This structure is treated as an effective dielectric layer characterized by an equivalent relative permittivity, denoted by $\varepsilon_{f2}$ (see Figure 1).

### 2.3. Diffuse Double-Layer Charge Density

The charges of bonded biomolecules will induce the diffusion of ions in the surrounding solution. Ions with the same sign as the charge on the HEMT surface will be repelled, creating a diffusion layer in the electrolyte. Conversely, ions of opposite sign will get attracted and create the so-called double layer. For the sake of clarity, we will provide a reminder of the diffuse charge expression here. For simplicity we propose that there is only one type of anion and cation ions in these regions which are monovalent and present in equal concentrations. This diffuse charge density ($Q_{diff}$) in the solution (beyond the functionalized layer) was defined from the Poisson equation solved in electrolyte (introduced in Gouy–Chapman theory), $Q_{diff}={2\varepsilon }_{f\_1}\frac{U_{T}}{l_{D}}sinh(\frac{\varphi_{l}}{{2U}_{T}})$, where $U_{T}$ is the thermal voltage, $l_{D}={\left(\varepsilon_{f\_1}U_{T}/2qn_{o}\right)}^{1/2}$ is the Debye screening length in the solution, $n_{o}$ is the concentration of ions in the solution, and $\varphi_{l}$ is the potential drop in the solution. The linearization of $sinh\left(\frac{\varphi_{l}}{{2U}_{T}}\right)$ term in $Q_{diff}$ is the commonly used approximation in sensing applications. After linearization, $Q_{diff}$ is expressed as

(3) $$Q_{diff}=\frac{\varepsilon_{f\_1}}{l_{D}}.$$

### 2.4. 2DEG Charge Density

**Figure 2:**
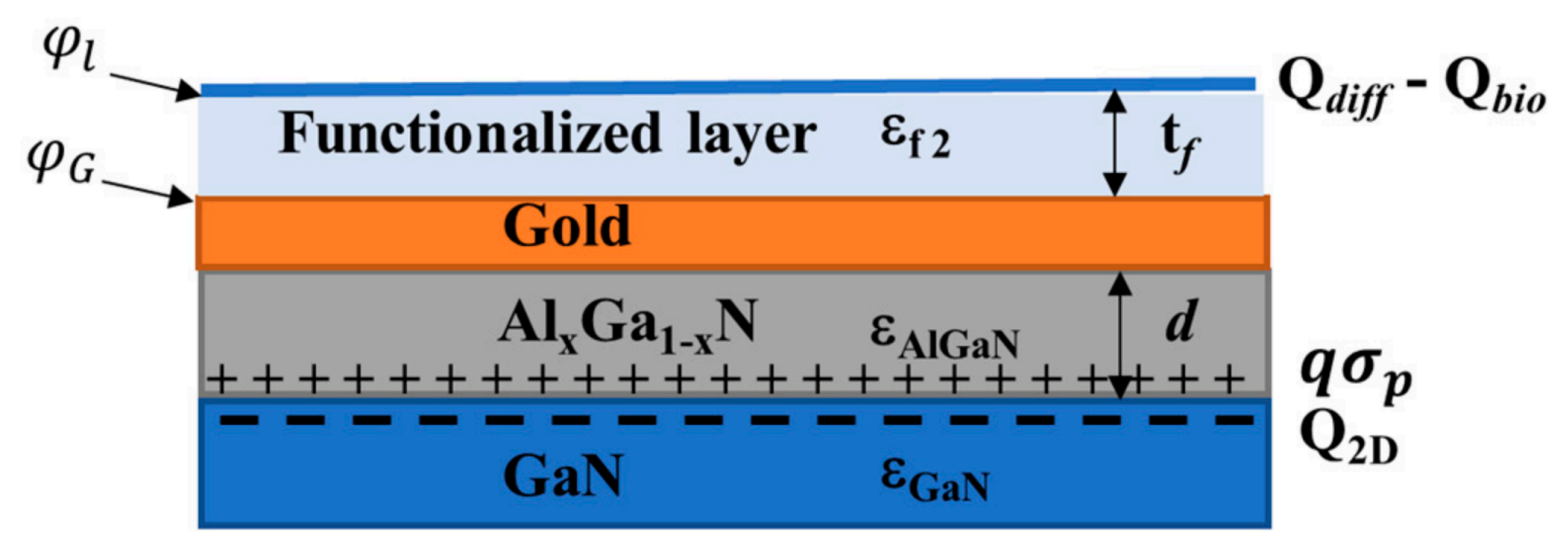
Schematic of the charge distribution in the GaN HEMT structure.

We consider an undoped Al_x_Ga_1-x_N/GaN heterostructure. The polarization-induced positive charge at the heterointerface gives rise to the formation of a 2DEG in the QW. The gold gate deposited on top of the AlGaN barrier layer (or on top of the insulating layer in the case of a MIS-HEMT) induces a Schottky barrier potential, $\phi_{Bn}$. In conventional HEMTs and MIS-HEMTs, the gate voltage is applied directly to the gold gate. In a biosensor configuration, however, the charges located above the functionalized layer modulate the QW charge density. By defining the gating potential on the gold plate as $\varphi_{G}$ (see Figure 2), the 2DEG charge density can be calculated explicitly as follows [43]:

(4) $$Q_{2D}(\varphi_{G},V_{ch})=qn_{2D1}f_{sm}+q(1-f_{sm})n_{2D2}$$

where $n_{2D1}=\frac{{(C}_{b}/q)(1-f_{1}\beta )V_{Go}}{1+{(C}_{b}/q)V_{Go}f_{1}}$, $\beta =D\gamma {\left({V_{Go}C}_{b}/q\right)}^{2/3}$, where $V_{Go}=\varphi_{G}-V_{off}-V_{ch}$ and $V_{off}=\phi_{Bn}-q\sigma_{p}/C_{b}-∆E_{C}/q$, and $f_{1}=3/\left(3DqV_{Go}+2\beta \right)$, $C_{b}=\varepsilon_{AlGaN}/d$ is the capacitance of AlGaN layer, $\sigma_{p}$ is the polarization-induced positive charge density, $f_{sm}=0.5+0.5tanh\left(V_{Go}/\left({\delta U}_{T}\right)\right)$, n2D2=D2DkT exp(gsUT) with $g_{s}=V_{Go}-q\theta_{sub}/C_{b}-\gamma {\left(\theta_{sub}\right)}^{\frac{2}{3}}/q$ and θsub=D kTlnexpVGo/UT+1 with $D_{2D}$ the two-dimensional density of states in QW in GaN, $\phi_{Bn}$ is the potential barrier height, $∆E_{C}$ is the conduction band offset and q is the elementary charge.

When the dielectric layer is incorporated between the gold plate and AlGaN layer, reverting HEMT to MIS-HEMT, the capacitance $C_{b}$ in (4) and (5) must be replaced by the effective capacitance $C_{eff}=\frac{C_{ins}C_{b}}{C_{ins}+C_{b}}$, where $C_{ins}$ is the capacitance of the insulating layer. For instance, AlNO can serve as an excellent gate dielectric for AlGAN/GaN [44].

### 2.5. Electrostatics of the System

Figure 2 presents the schematic distribution of charges in the GaN HEMT biosensor. The charge neutrality of the system assumes

(5) $$Q_{diff}-Q_{bio}-Q_{2D}+q\sigma_{p}=0$$

Here, we assume that the functionalized surface suppresses or makes negligible the contribution of additional interfacial charge mechanisms, such as specific ion adsorption and pH-dependent surface protonation. If such effects are significant, the corresponding charge terms can be included in Equation (5) and treated using the approach presented in [42].

Assuming that $V_{G}$ is the effective voltage applied to the reference electrode and that $\varphi_{l}$, introduced above, represents the potential drop across the analyte solution, Gauss’s law yields

(6) $$C_{f2}(V_{G}+\varphi_{l}-\varphi_{G})=Q_{diff}-Q_{bio}$$

where $C_{f2}=\varepsilon_{f2}/t_{f}$, and $\varepsilon_{f2}$ and $t_{f}$ are the dielectric permittivity and thickness of the functionalized layer, respectively. For simplicity we define ${\varphi_{G}}^{*}=\varphi_{G}-V_{G}$. By substituting (3) into (6), we have

(7) $${\varphi_{l}}^{*}=\left(Q_{bio}-C_{f2}{\varphi_{G}}^{*}\right)/\left(\frac{\varepsilon_{f_{1}}}{l_{D}}-C_{f2}\right).$$

By substituting (4), (6), and (7) into (5), the gating potential (${\varphi_{G}}^{*}$) can be determined self-consistently for a given set of system parameters:

(8) $$C_{f2}\left(\left(Q_{bio}-C_{f2}{\varphi_{G}}^{*}\right)/\left(\frac{\varepsilon_{f_{1}}}{l_{D}}-C_{f2}\right)-{\varphi_{G}}^{*}\right)-Q_{2D}({\varphi_{G}}^{*})+q\sigma_{p}=0$$

### 2.6. Drain Current of the GaN HEMT Biosensor

We use the drain current equation developed for GaN HEMT [45],

(9) $$I_{D}=I_{s}{\left.\left(\frac{Q_{2D}(\varphi_{G},V_{ch})}{2C_{b}U_{T}}-{\left(\frac{Q_{2D}(\varphi_{G},V_{ch})}{2C_{b}U_{T}}\right)}^{2}\right)\right|}_{V_{ch}=V_{S}}^{V_{ch}=V_{D}}$$

where $I_{s}=2\mu C_{eff}{U_{T}}^{2}W/L$ is the specific current, μ is the electron mobility, and W and L are device dimensions. Here $Q_{2D}(\varphi_{G},V_{ch})$ is defined in (4) and $\varphi_{G}$ can be calculated from (8).

### 2.7. Algorithm for Determining the Target Molecule Concentration, $c_{o}$

Assume that the biosensor measures the drain current ($I_{D})$. Using (9), the corresponding 2DEG charge density $Q_{2D}$ is determined first. Then, for the given drain and reference electrode voltages, the gating potential ($\varphi_{G}$) is obtained from (4). Finally, the biomolecular charge $Q_{bio}\left(t\right)$ is determined from the charge neutrality using Equation (8) for the corresponding time instant. The target molecule concentration, $c_{o}$, is then obtained from (1).

## 3. Discussion and Validation

To investigate the performance of the GaN HEMT biosensor and to validate the accuracy of the developed analytical model, comprehensive simulations were performed using COMSOL Multiphysics 6.2. The simulation framework couples the semiconductor and electrostatics interfaces to model the electrical behavior of the GaN HEMT and its interaction with the electrolyte solution. The Transport of Diluted Species interface was employed to describe the transport of ionic species in the electrolyte, accounting for diffusion, migration under the electric field, and convection. Biomolecular binding at the functionalized sensing surface was modeled using the Surface Reactions interface. To ensure the continuity of the electric displacement vector across the semiconductor–electrolyte interface, Global ODEs and DAEs were introduced. Finally, the Multiphysics coupling was used to self-consistently solve the coupled semiconductor transport, electrostatic, and chemical transport equations, ensuring consistent interactions between charge distributions, electric potentials, and biochemical processes throughout the device.

### 3.1. Validation of COMSOL Simulations with Experimental Data

**Figure 3:**
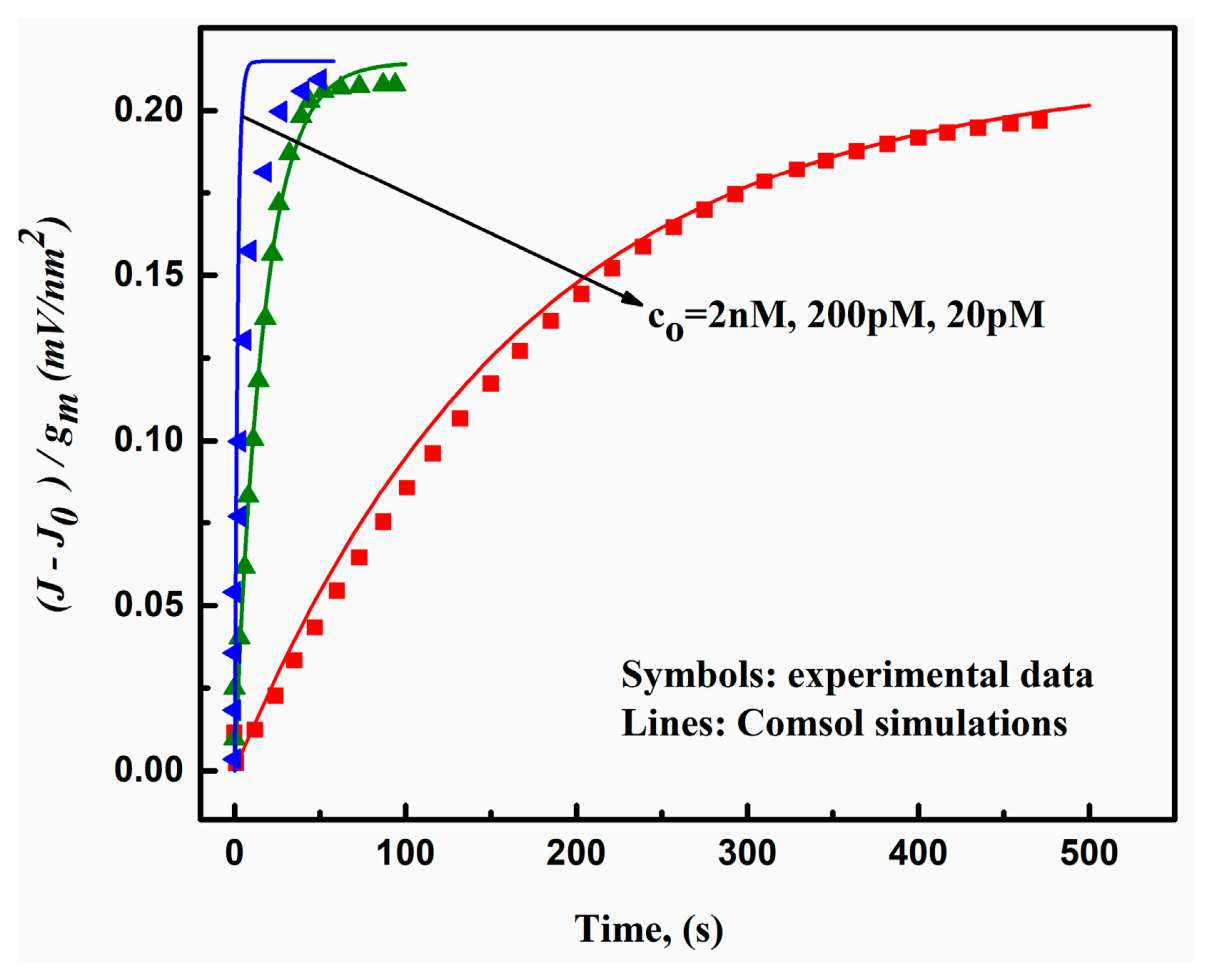
The sensitivity simulated with COMSOL Multiphysics and extracted from [39].

**Figure 4:**
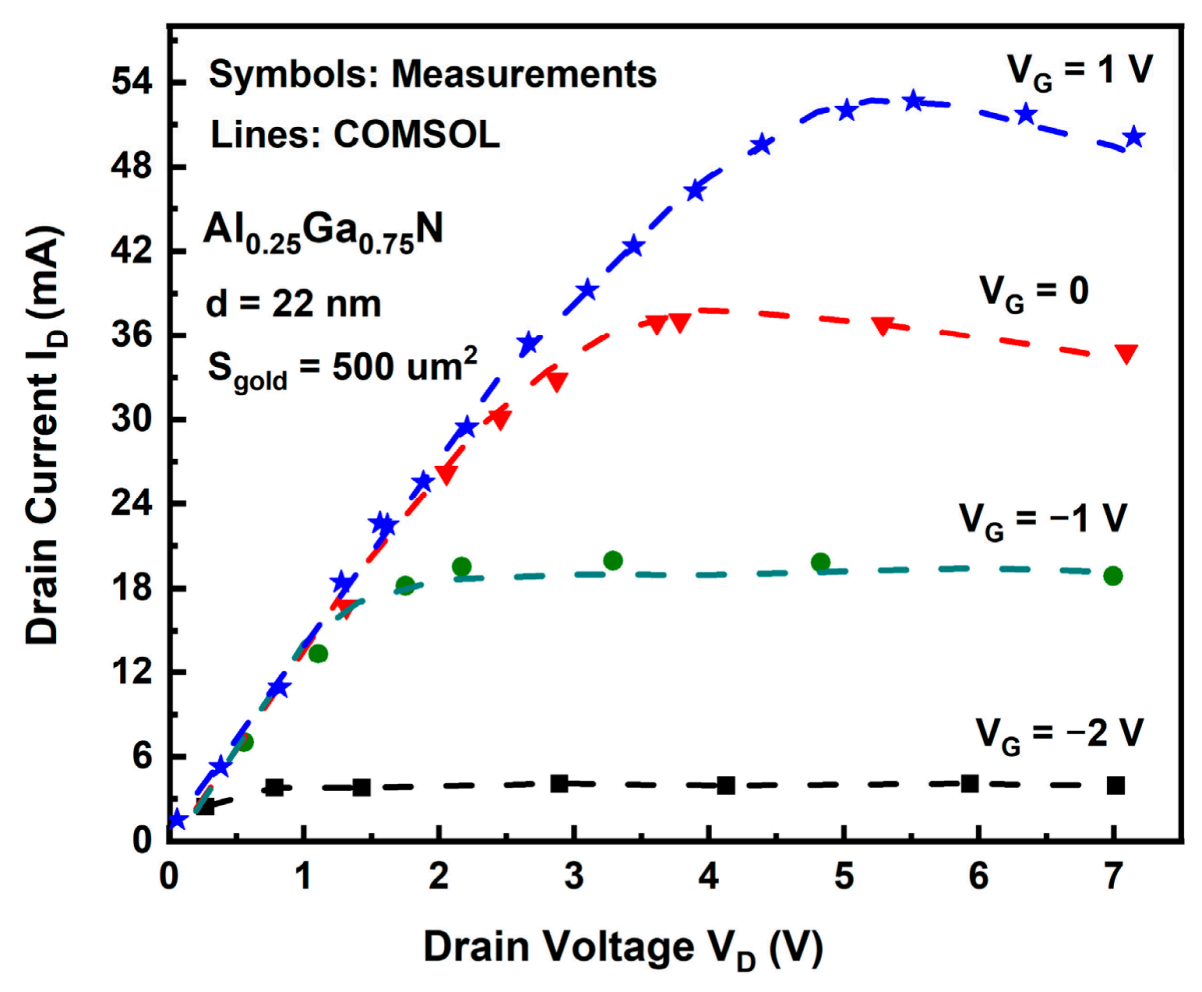
Comparison of the simulated and experimental output characteristics of the GaN HEMT at different gate voltages. Experimental data are from [2].

To ensure the accuracy of the COMSOL simulations, the numerical model was first validated against experimental data. The most challenging aspect of the simulation is the modeling of biomolecular binding kinetics at the sensor surface. To calibrate the Surface Reactions interface, we used the experimental data reported in [39], where the binding kinetics of the biotin–streptavidin system was characterized. This study was selected because it provides comprehensive kinetic measurements over a wide range of analyte concentrations and association times. The adsorption and desorption rate constants were taken directly from [39], with k_ads_ = 5 × 10^8^ M^−1^s^−1^ and k_des_ = 8 × 10^−5^ M^−1^ s^−1^, respectively. A fluid flow velocity of 5 µm·s^−1^ was also adopted. The surface charge density of a single bound streptavidin molecule was assumed to be $q_{mol}=-17\;mC/m^{2}$, while the effective binding diameter was set to 5 nm. A detailed comparison between the experimental measurements and the COMSOL simulations was previously presented in [42]; for completeness, the results are also shown here. As illustrated in Figure 3, excellent agreement is achieved over a broad concentration range (2–200 pM), confirming the accuracy of the adopted COMSOL modeling approach. In addition, Figure 3 highlights that the time required for the sensor response to approach saturation strongly depends on the analyte concentration, providing useful guidance for selecting the optimal measurement time for different concentration ranges. The electrical characteristics of the simulated GaN HEMT were further validated against the experimental measurements reported in [2], as shown in Figure 4. To accurately reproduce the measured drain current characteristics, the Caughey–Thomas mobility model was implemented in the semiconductor module of COMSOL simulations to account for the electric-field-dependent carrier mobility. The simulated output characteristics ($I_{D}-V_{D}$) were compared with the data from [2] for different gate voltages. As illustrated in Figure 4, excellent agreement is achieved in both the linear and saturation operating regions. The model accurately captures the transition from the linear to the saturation regime as well as the dependence of the drain current on the gate bias, demonstrating that the adopted simulation framework reliably describes the electrical behavior of the GaN HEMT and is suitable for subsequent biosensing simulations.

### 3.2. Sensitivity Analysis of the GaN HEMT Operating Regimes

**Figure 5:**
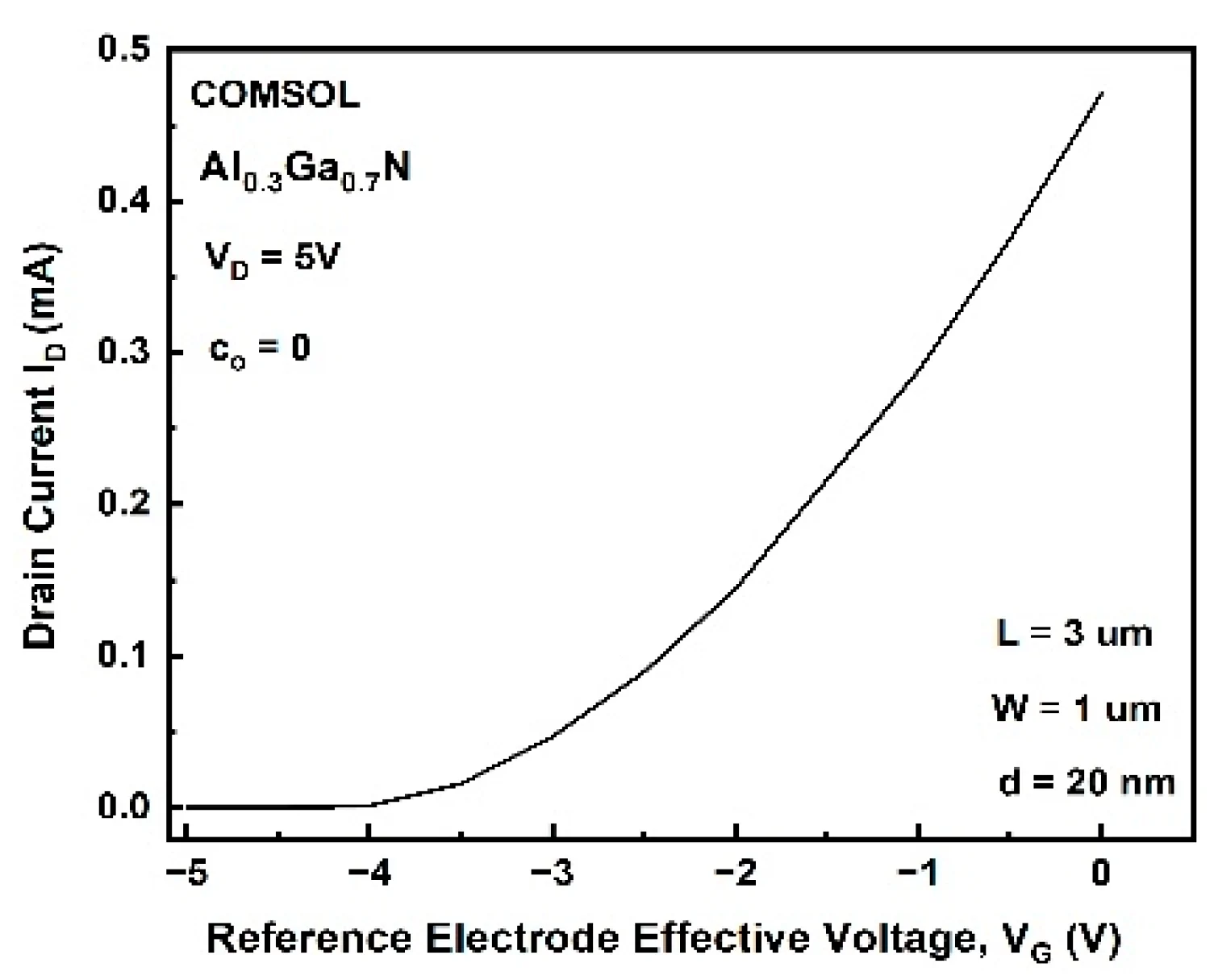
Transfer characteristics of GaN HEMT biosensor at the absence of biomolecules.

Studies on Si-based FET sensors have shown that the channel becomes extremely sensitive in the depletion regime [46,47]. Since the GaN HEMT is a normally on device, a negative gate voltage must be applied to drive it into the depletion regime. In our COMSOL simulations, a reference electrode is immersed in the solution with a gate voltage ($V_{G})$ applied to it. The ionic concentration of the solution is set to 100 mol/m^3^, representing near-physiological electrolyte conditions. The transfer characteristic of the hybrid system at zero concentration of target biomolecules is shown in Figure 5.

**Figure 6:**
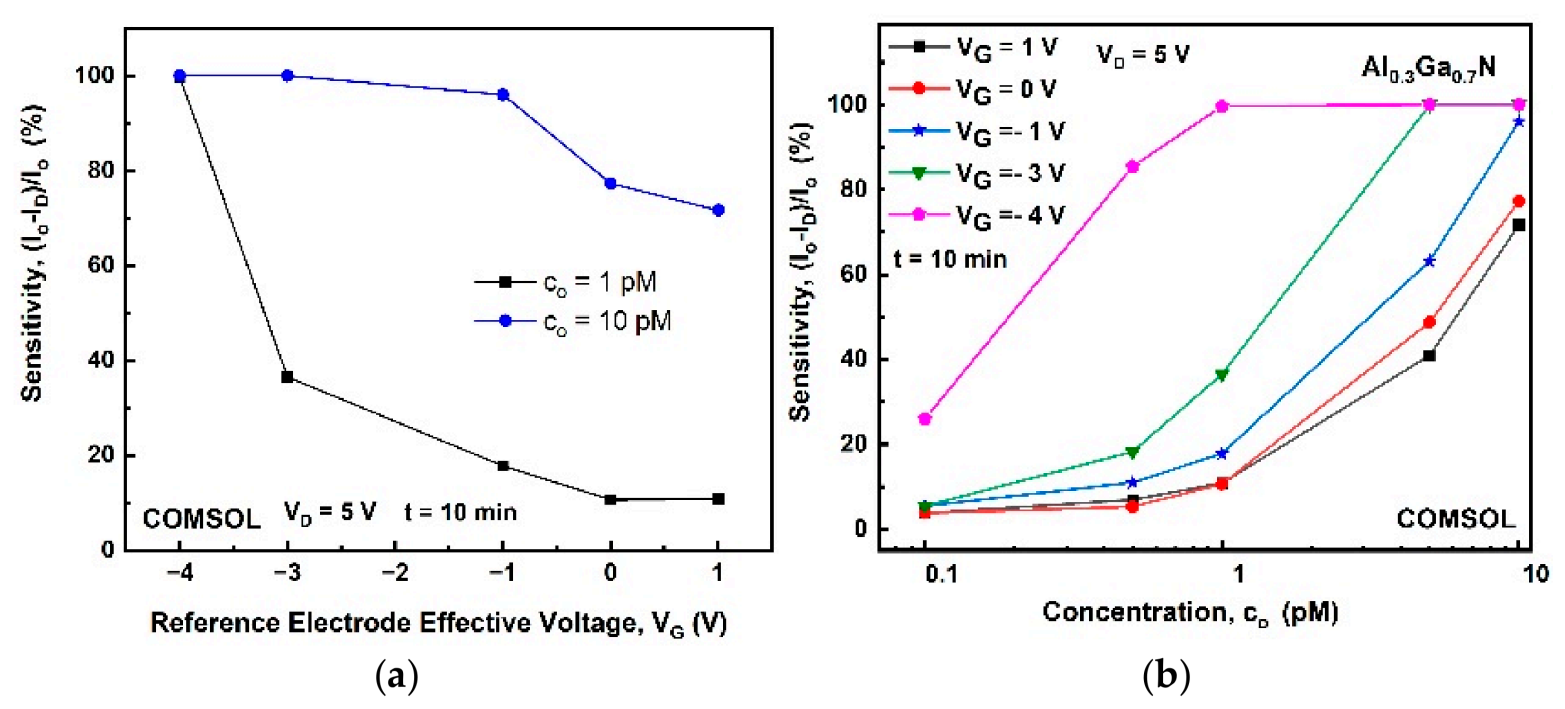
Relative drain current sensitivity (**a**) versus effective gate voltage for 1 pM and 10 pM concentrations, and as a function of target biomolecule concentration for different effective gate voltages (**b**).

Throughout all simulations presented in this work, the solution pH is fixed at 7.4, corresponding to physiological conditions. Furthermore, we assume that the isoelectric point of the target biomolecule is lower than the solution pH, resulting in a negatively charged surface. This means that the GaN HEMT will operate in the depletion regime. Figure 6 shows the relative sensitivity extracted from the COMSOL simulations, which is defined as $\left(I_{o}-I_{D}\right)/I_{o}$, where $I_{o}$ is the drain current in the absence of biomolecule binding at the corresponding applied gate voltage, and $I_{D}$ is the drain current after biomolecule binding. We use the same binding kinetics of the biotin–streptavidin system as described above for Figure 3. As shown in Figure 6a, for a biomolecule concentration of 10 pM, the sensor exhibits a high relative sensitivity of nearly 80% over a wide range of gate voltages. In contrast, at a concentration of 1 pM, high sensitivity can only be achieved when the device operates in the subthreshold regime. As seen in Figure 6b, at a concentration of 0.1 pM, the relative sensitivity is almost negligible for gate voltages above −3 V but increases to approximately 24% when the gate voltage is reduced to −4 V. However, operation in the subthreshold regime also has limitations. At high concentrations of target biomolecules in the solution, the drain current decreases dramatically and may approach the noise floor, making reliable detection difficult. Therefore, the optimal operating regime should be selected according to the target concentration range. For low-concentration detection, subthreshold operation provides enhanced sensitivity, whereas operation above threshold voltage ($V_{th}$) is more suitable for detecting higher analyte concentrations while maintaining sufficient output current. Table 1 summarizes our observations.

### 3.3. Validation of Analytical Model

**Figure 7:**
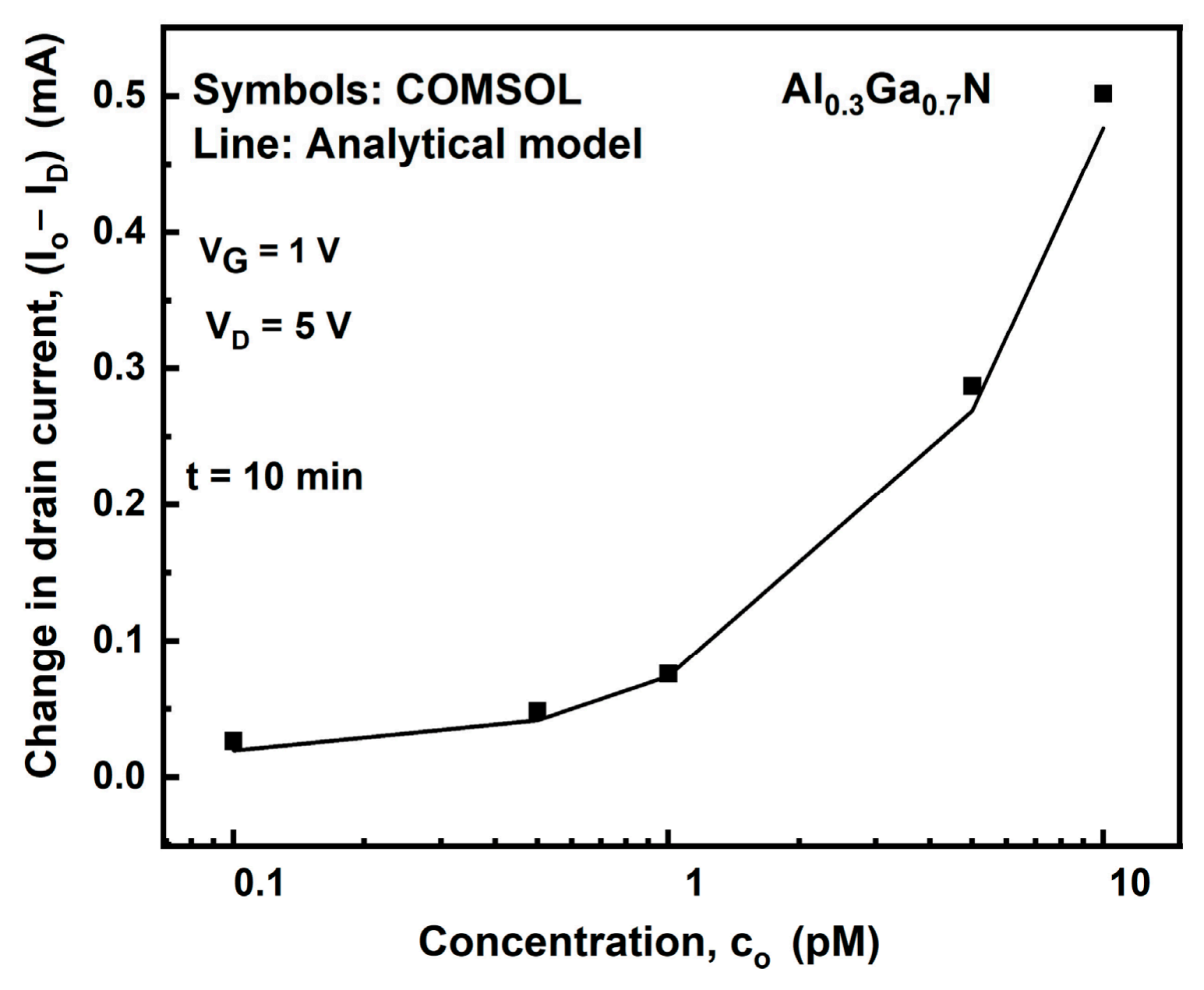
Drain current variation as a function of analyte concentration, calculated using the analytical model and COMSOL simulations.

**Figure 8:**
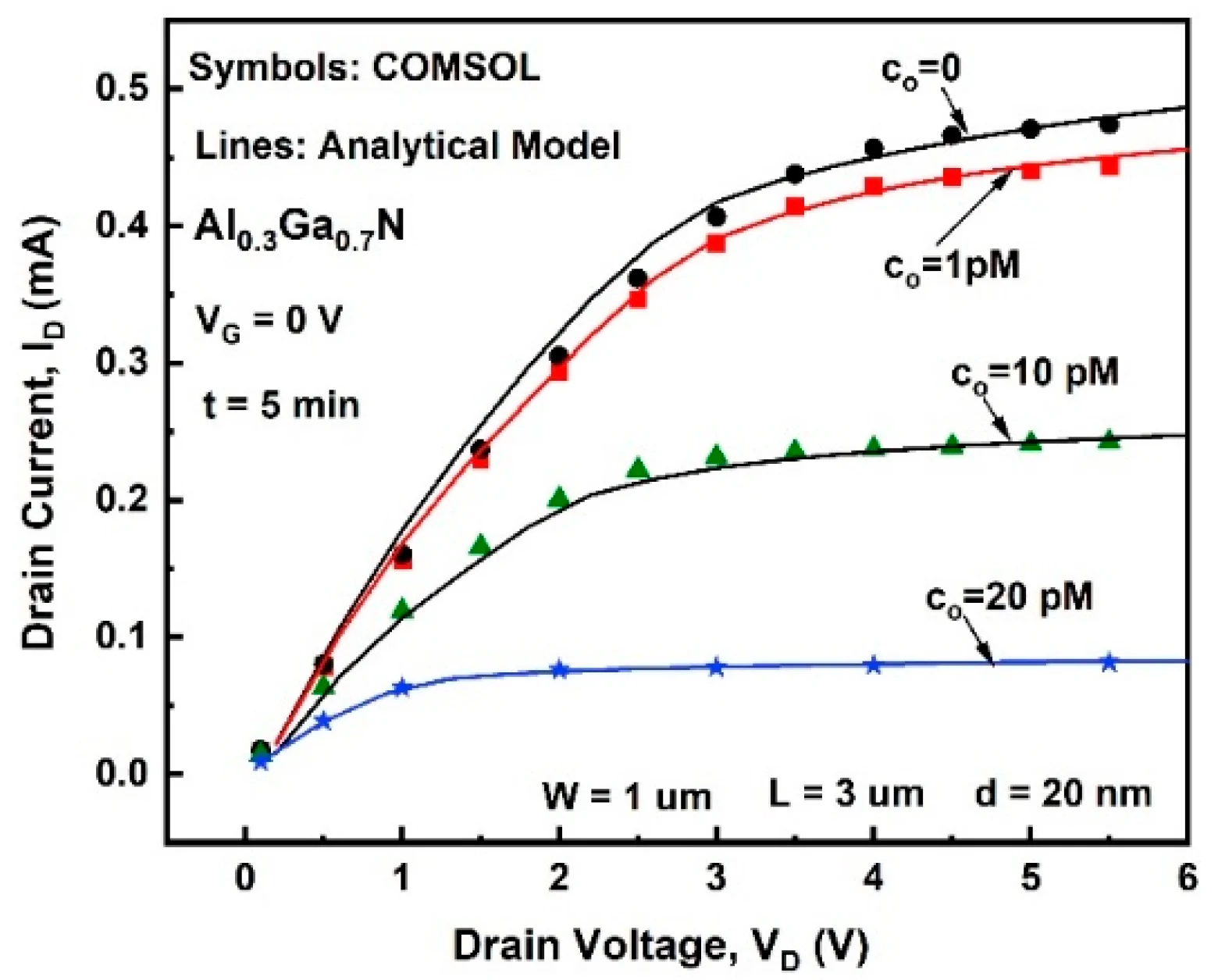
Comparison of volt–ampere characteristics at different gating potentials induced by variations in analyte concentration, obtained from the analytical model and COMSOL simulations.

To validate the developed analytical model of the GaN HEMT biosensor, its predictions were compared with COMSOL Multiphysics simulation results. First, the analyte concentration was swept, and the corresponding change in the drain current was calculated. The comparison between the analytical model and the numerical simulations is presented in Figure 7. As can be seen, the analytical model accurately reproduces the drain current variation over the investigated concentration range, demonstrating its capability to predict the electrical response of the biosensor to biomolecule binding. A further validation is provided in Figure 8, which compares the drain current–drain voltage characteristics at different gating potentials induced by variations in analyte concentration. The analytical model shows excellent agreement with the COMSOL simulations over the entire operating range, confirming that it accurately captures the influence of analyte-induced surface potential changes on the device output characteristics.

Our calculations for the streptavidin–biotin system, presented in Figure 7, show that sensitivity dramatically decreases below 1 pM. By assuming a perfectly noiseless environment, we can estimate the lowest theoretical limit of detection (LOD) by setting a threshold at which a valid detection event requires a drain current shift of $\Delta I_{D}=0.1$ mA. The LOD strongly depends on the target analyte, surface functionalization, and device architecture. When the device architecture and functionalization are kept fixed, the LOD is determined by the analyte’s net molecular charge (controlled by the solution pH) and its adsorption coefficient. For the system described above, the LOD is 1 pM. This threshold may vary for other antigen–antibody pairs depending on their specific kinetic and electrical properties. The presented model is design-oriented and allows the LOD to be calculated based on the system parameters.

## 4. Conclusions

This work presents a comprehensive, physics-based analytical model for GaN HEMT and MIS-HEMT biosensors, directly linking biomolecular recognition events to the transistor’s electrical response. Because the analytical expressions are explicit, they permit a direct investigation of the effects of electrical, biochemical, and structural parameters on sensor characteristics, guiding the optimization of electrostatic coupling. The analytical model accurately reproduces numerical simulation results. Additionally, we establish optimal sensor-biasing strategies relative to target biomolecule concentrations, providing practical guidelines to maximize performance across various concentration regimes. Finally, owing to its compact mathematical formulation, the model can be seamlessly integrated into circuit-level simulators for the design and optimization of next-generation biosensing systems.

## Figures and Tables

**Table 1:** Recommended reference electrode bias operating regimes for different target biomolecule concentration ranges.

Sub-Picomolar Range	Picomolar Range	Picomolar-to-Nanomolar Range
$V_{G}<V_{th}$	VG=0 V	VG≈1 V *

* Assuming a Schottky barrier height of approximately 1.1 eV.

## Data Availability

The original contributions presented in this study are included in the article. Further inquiries can be directed to the corresponding author.

## References

[1] Revathy A., Ravi S., Narayana A.L., Devi K.N., Pandurangan R. (2025). Advanced gallium nitride high electron mobility transistors for biosensing applications: Progress, challenges, and future perspectives. Microelectron. Eng..

[2] Mishra S., Kachhawa P., Mondal P., Ghosh S., Tripura C., Chaturvedi N. (2022). AlGaN/GaN HEMT based biosensor for detection of the HER2 antigen spiked in human serum. IEEE Trans. Electron Dev..

[3] Peesa R.B., Kumar P.M., Panda D.K. Simulation of GaN MOS-HEMT based bio-sensor for breast cancer detection. Proceedings of the First Annual Conference on Computer-Aided Developments in Electronics and Communication (CADEC-2019).

[4] Chen K.H., Kang B.S., Wang H.T., Lele T.P., Ren F., Wang Y.L., Chang C.Y., Pearton S.J., Dennis D.M., Johnson J.W. (2008). C-erbB-2 sensing using AlGaN/GaN high electron mobility transistors for breast cancer detection. Appl. Phys. Lett..

[5] Chang C.W., Chen P.H., Wang S.H., Hsu S.Y., Hsu W.T., Tsai C.C., Wadekar P.V., Puttaswamy S., Cheng K.H., Hsieh S. (2018). Fast detection of tumor marker CA 19-9 using AlGaN/GaN high electron mobility transistors. Sens. Actuators B Chem..

[6] Li J.D., Cheng J.J., Miao B., Wei X.W., Xie J., Zhang J.C., Zhang Z.Q., Li H.W., Wu D.M. (2015). Label free electrical detection of prostate specific antigen with millimeter grade biomolecule-gated AlGaN/GaN high electron mobility transistors. Microsyst. Technol..

[7] Kang B.S., Wang H.T., Lele T.P., Tseng Y., Ren F., Pearton S.J., Johnson J.W., Rajagopal P., Roberts J.C., Piner E.L. (2007). Prostate specific antigen detection using AlGaN/GaN high electron mobility transistors. Appl. Phys. Lett..

[8] Pulikkathodi A.K., Sarangadharan I., Hsu C.P., Chen Y.H., Hung L.Y., Lee G.Y., Chyi J.I., Lee G.B., Wang Y.L. (2018). Enumeration of circulating tumor cells and investigation of cellular responses using aptamer-immobilized AlGaN/GaN high electron mobility transistor sensor array. Sens. Actuators B Chem..

[9] Repaka L., Ajayan J., Bhattacharya S., Mounika B., Akshaykranth A., Nirmal D. (2025). A review of microelectronic AlGaN/GaN HEMT biosensors for the detection of various cancer diseases and bacterial/viral pathogens. Microsyst. Technol..

[10] Kang B.S., Wang H.T., Ren F., Pearton S.J. (2008). Electrical detection of biomaterials using AlGaN/GaN high electron mobility transistors. J. Appl. Phys..

[11] Poonia R., Bhargava L., Bhat A.M., Periasamy C. (2024). Recessed trench gate AlGaN/GaN HEMT for pH monitoring: Design and sensitivity evaluation. IEEE Trans. Nanotechnol..

[12] Varghese A., Periasamy C., Bhargava L., Dolmanan S.B., Tripathy S. (2019). Linear and circular AlGaN/AlN/GaN MOS-HEMT-based pH sensor on Si substrate: A comparative analysis. IEEE Sens. Lett..

[13] Poonia R., Periasamy C., Bhat A.M., Bhargava L., Sahu C. (2024). Performance assessment of AlGaN/GaN HEMT for human serum albumin detection using charge deduction methodology. IEEE Sens. J..

[14] Thakur R.R., Kc S., Mishra S., Taliyan R., Chaturvedi N. (2024). Bio-Interface Analysis and Detection of A β using GaN HEMT-based Biosensor. J. Electrochem. Soc..

[15] Thakur R.R., Saini A.K., Taliyan R., Chaturvedi N. (2024). Meander-gated dual cap GaN HEMT-based portable noninvasive COVID-19 detection platform. Appl. Phys. Lett..

[16] Sarangadharan I., Regmi A., Chen Y.W., Hsu C.P., Chen P.C., Chang W.H., Lee G.Y., Chyi J.I., Shiesh S.C., Lee G.B. (2018). High sensitivity cardiac troponin I detection in physiological environment using AlGaN/GaN High Electron Mobility Transistor (HEMT) Biosensors. Biosens. Bioelectron..

[17] Hu S., Zou Y., Jiang X., Weng Y., Liu Y., Tang X., Yang G., Lu N. (2025). A high electron mobility transistor biosensor-based GaN for facile and sensitive detection of *Staphylococcus aureus*. Microchem. J..

[18] Son K.A., Yang B., Liao A., Moon J., Prokopuk N. High-Sensitivity GaN Microchemical Sensors. NASA Tech Briefs, October 2009. http://www.techbriefs.com/component/content/article/5788.

[19] Woo K., Kang W., Lee K., Lee P., Kim Y., Yoon T.S., Cho C.Y., Park K.H., Ha M.W., Lee H.H. (2020). Enhancement of cortisol measurement sensitivity by laser illumination for AlGaN/GaN transistor biosensor. Biosens. Bioelectron..

[20] Zeggai O., Ould-Abbas A., Bouchaour M., Zeggai H., Sahouane N., Madani M., Trari D., Boukais M., Chabane-Sari N.E. (2014). Biological detection by high electron mobility transistor (HEMT) based AlGaN/GaN. Phys. Status Solidi (c).

[21] Lee H.H., Bae M., Jo S.H., Shin J.K., Son D.H., Won C.H., Lee J.H. (2016). Differential-mode HEMT-based biosensor for real-time and label-free detection of C-reactive protein. Sens. Actuators B Chem..

[22] Hemaja V., Panda D.K. (2021). Dielectric modulated enhancement mode N-polar GaN MIS-HEMT biosensor for label free detection. ECS J. Solid State Sci. Technol..

[23] Mouffoki F., Bouguenna D., Dahou F.Z., Beloufa A., Loan S.A. (2022). Performance evaluation of electrical properties of GaN MOS-HEMTs based biosensors for rapid detection of viruses. Mater. Today Commun..

[24] Liu Y., Ma Y., Guo H., Fu S., Liu Y., Wei G., Liu Y., Hao Y., Chen D. (2024). The study of N-polar GaN/InAlN MOS-HEMT and T-gate HEMT biosensors. J. Phys. D Appl. Phys..

[25] Kachhawa P., Mishra S., Jain A.K., Tripura C., Joseph J., Radha V., Chaturvedi N. (2022). Antigen-antibody interaction-based GaN HEMT biosensor for C3G detection. IEEE Sens. J..

[26] Hemalatha R.G., Kumar M.A., Mishra G.S., N M.K., Ismail K.B., Mahalingam S., Kim J. (2025). Design and Simulation of advanced boron-doped GaN cap layer on AlGaN/GaN MOSHEMTs for enhanced label-free biosensing applications. Biomed. Microdevices.

[27] Shaveta, Ahmed H.M., Chaujar R. (2020). Rapid detection of biomolecules in a dielectric modulated GaN MOSHEMT. J. Mater. Sci. Mater. Electron..

[28] Varghese A., Periasamy C., Bhargava L. (2019). Fabrication and charge deduction based sensitivity analysis of GaN MOS-HEMT device for glucose, MIG, C-erbB-2, KIM-1, and PSA detection. IEEE Trans. Nanotechnol..

[29] Kumar A., Varghese A., Kalra D., Pancholi S., Sharma G.K. (2023). Optimizing bio-sensor design with support vector regression technique for AlGaN/GaN MOS–HEMT. IEEE Sens. Lett..

[30] Sriramani P., Mohankumar N., Durai L., Prasamsha Y., Rakesh N. (2025). Analytical model for DG-AlGaN/GaN MOS-HEMT for sensitive analysis of pH analytes and charged biomolecules. Sens. Int..

[31] Chaturvedi N., Singh K., Kachhawa P., Lossy R., Mishra S., Chauhan A., Kharbanda D.K., Jain A.K., Thakur R.R., Saxena D. (2020). AlGaN/GaN HEMT based sensor and system for polar liquid detection. Sens. Actuators A Phys..

[32] Huq H., Trevino I.I.H., Castillo J. (2016). Characteristics of AlGaN/GaN HEMTs for detection of MIG. J. Mod. Phys..

[33] Singh Y., Gupta N., Charaya N., Kumar A., Kanungo V. (2026). AlGaN/GaN HEMT Biosensors for Next-Generation Applications: A State-of-the-Art Review. Appl. Biochem. Biotechnol..

[34] Li C., Chen X., Wang Z. (2024). Review of the AlGaN/GaN high-electron-mobility transistor-based biosensors: Structure, mechanisms, and applications. Micromachines.

[35] Fauzi N., Mohd Asri R.I., Mohamed Omar M.F., Manaf A.A., Kawarada H., Falina S., Syamsul M. (2023). Status and prospects of heterojunction-based HEMT for next-generation biosensors. Micromachines.

[36] Hu S., Jiang X., Yang L., Tang X., Yang G., Hu Y., Wang J., Lu N. (2023). A miniature biomedical sensor for rapid detection of Schistosoma Japonicum antibodies. Biosensors.

[37] Yang X., Ao J., Wu S., Ma S., Li X., Hu L., Liu W., Han C. (2020). Low-power pH sensor based on narrow channel open-gated Al0. 25Ga0. 75N/GaN HEMT and package integrated polydimethylsiloxane microchannels. Materials.

[38] Falina S., Syamsul M., Rhaffor N.A., Sal Hamid S., Mohamed Zain K.A., Abd Manaf A., Kawarada H. (2021). Ten years progress of electrical detection of heavy metal ions (HMIs) using various field-effect transistor (FET) nanosensors: A review. Biosensors.

[39] Duan X., Li Y., Rajan N.K., Routenberg D.A., Modis Y., Reed M.A. (2012). Quantification of the affinities and kinetics of protein interactions using silicon nanowire biosensors. Nat. Nanotechnol..

[40] Hibbert D.B., Gooding J.J., Erokhin P. (2002). Kinetics of irreversible adsorption with diffusion: Application to biomolecule immobilization. Langmuir.

[41] Squires T.M., Messinger R.J., Manalis S.R. (2008). Making it stick: Convection, reaction and diffusion in surface-based biosensors. Nat. Biotechnol..

[42] Yesayan A., Grabski A., Jazaeri F., Sallese J.M. (2025). Design-Oriented Analytical Model for Nanowire Biosensors Including Dynamic Aspects. Trans. Electron Devices.

[43] Yesayan A., Jazaeri F., Sallese J.-M. (2026). Explicit Charge-Based Current Model of MIS-HEMT. J. Comput. Electron..

[44] Wang Q., Cheng X., Zheng L., Ye P., Li M., Shen L., Li J., Zhang D., Gu Z., Yu Y. (2017). Band alignment between PEALD-AlNO and AlGaN/GaN determined by angle-resolved X-ray photoelectron spectroscopy. Appl. Surf. Sci..

[45] Jazaeri F., Sallese J.M. (2019). Charge-based EPFL HEMT model. IEEE Trans. Electron Devices.

[46] Yesayan A., Jazaeri F., Sallese J.M. (2020). Analytical modeling of double-gate and nanowire junctionless ISFETs. Trans. Electron Devices.

[47] Chen C.W., Lin R.Z., Chiang L.C., Pan F.M., Sheu J.T. (2019). Junctionless gate-all-around nanowire field-effect transistors with an extended gate in biomolecule detection. Jpn. J. Appl. Phys..

